# Supplementary figures and images for: Post-Injury Buprenorphine Administration Is Associated with Long-Term Region-Specific Glial Alterations in Rats
Source: Pharmaceutics. 2022 Sep 28;14(10):2068. doi: 10.3390/pharmaceutics14102068 (PMC9607339; doi:10.3390/pharmaceutics14102068)

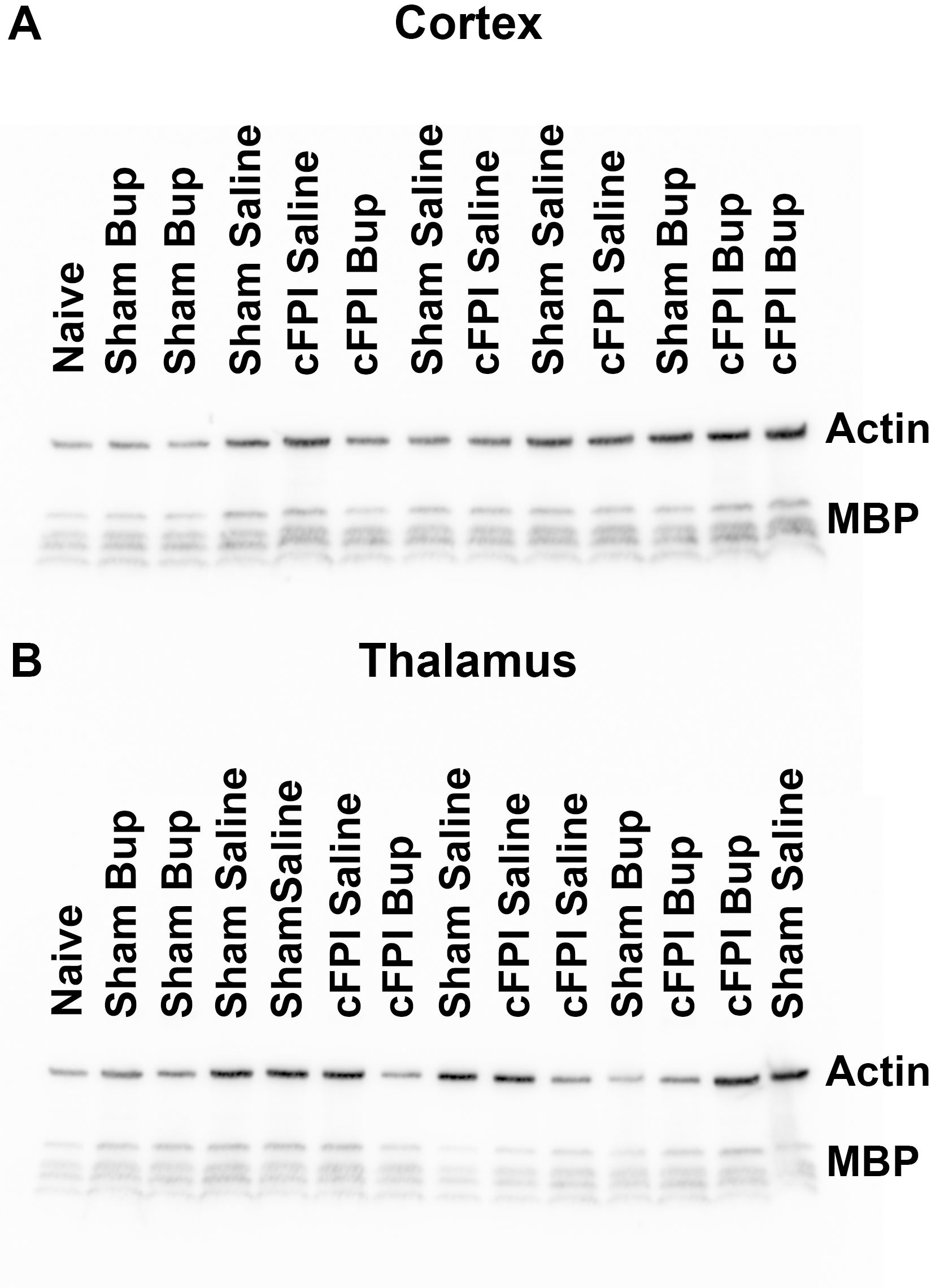

Supplement: Supplementary file 1 [file pharmaceutics-14-02068-s001.zip › Figure S1.jpg]
